# Trends of patients’ visit at the University Dental Hospital prior to and during the COVID-19 pandemic: a cross-sectional study

**DOI:** 10.11604/pamj.2023.44.87.37372

**Published:** 2023-02-14

**Authors:** Halima Leruk Darak, Olivia Awino Osiro, Desmond Otieno K'Owino, Bernard Nzioka Mua

**Affiliations:** 1 Department of Dental Sciences, University of Nairobi Dental Hospital, Nairobi, Kenya

**Keywords:** Dental education, oral diagnosis, dental clinics, SARS-CoV-2, healthcare quality

## To the editors of the Pan African Medical Journal

To contain the spread of COVID-19 in Kenya, enforced measures included closure of learning institutions, a ban on gatherings, compulsory wearing of protective face masks in public places and imposition of nationwide nighttime curfews. Moreover, there was cessation of movements in and out of areas considered high risk such as the capital city, Nairobi and the surrounding conurbation [1]. Oral health service providers were at greater risk of infection because of proximity to patients while working and exposure to aerosols, blood, contaminated instruments and splatter during routine procedures. Moreover, this risk extended to patients and administrative staff within a confined surgery and waiting area. Although, cross-infection prevention and control measures were routinely practiced in the dental setting in Kenya even before the pandemic [2], the Ministry of Health issued interim guidelines for dental practice such as: reducing service provision to dental emergencies; cancelling non-emergency appointments for vulnerable groups like pregnant women, children, the elderly and those with underlying medical co-morbidities; postponement of non-urgent and elective procedures for the general population; and tele-screening [1].

The University of Nairobi Dental Hospital is an easily accessible and affordable teaching hospital located in Nairobi, Kenya and offers various specialist oral health services to the public. Through a census and an analytical cross-sectional study, we aimed to describe the trends in new patient visits by comparing characteristics of those who visited the hospital prior to and during the pandemic, testing the hypothesis that differences were observed between the two periods. Following the announcement of the first case of COVID-19 in Nairobi on 12^th^ March 2020 and ensuing government directives [3], all campuses of the University of Nairobi, including the affiliated hospital, were closed for physical learning; therefore, we defined the pre-pandemic period from 1^st^ June 2019 up to this point. We considered the pandemic period from April 2020 to July 2021; however, the hospital resumed operations on 17^th^ August, thus there were no new patients registered between 13^th^ March and 16^th^ August 2020. This justified the selection of the two-year period up to July 2021 to enable comparison of at least eight months of normal operations both before and during the pandemic. From the digital filing system, sociodemographic data of patients were summarized including age, gender and residence. Additionally, the first clinic attendance was tallied. Through systematic random sampling, every third record of 312 patient files were analyzed for presenting complaints; 156 files each of patients who visited prior to and during the pandemic. Data was verified to have all required information as per the facility´s standard operating procedure for patient registration. The few missing entries were retrieved from the relevant patient´s file and completed. Descriptive summaries were presented in frequencies, percentages, means and mode. For hypothesis testing, data were subjected to χ^2^, Wilcoxon-Rank and student t-test at α=0.05. Ethical approval was obtained from the Kenyatta National Hospital/University of Nairobi Ethics and Research Committee (UP971/12/2021). Waiver of consent and authorization to utilize patient records was granted. Patient confidentially was ensured by blocking out any personal identifiable information.

**Figure 1:**
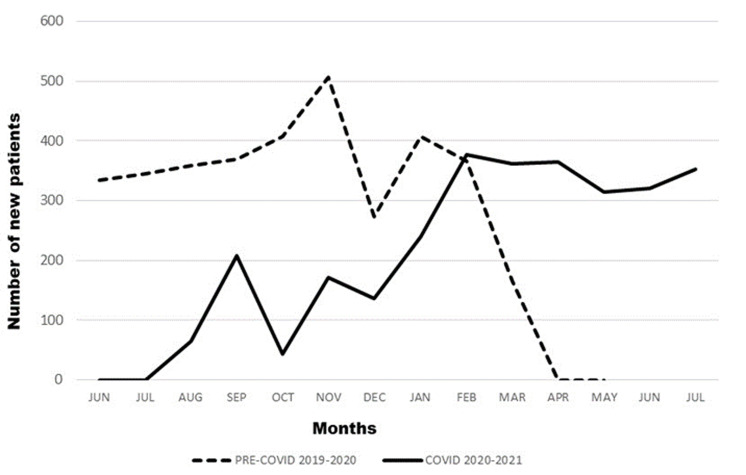
pattern of new patients’ visit at the University of Nairobi Dental Hospital prior to and during the pandemic

The total number of new registered patients was 6,486. Of these, 3,529 visited before the pandemic while 2,957 visited during the pandemic, translating to an overall 8.6% decrease in new patients seen during the pandemic. Figure 1 summarizes the trend in new patient visits at the hospital. The mean monthly visits prior to the pandemic were 353.2±89.5 while they reduced to 251.08±113.95 during the pandemic (t=2.15, p=0.0428). Although the modal age group remained the same during both periods (21-30 years), prior to the pandemic, the second-commonest age group visiting was ≤10 years unlike during the pandemic when the second-commonest age group was 31-40 years. Wilcoxon Signed-Rank test showed a significant difference in the age group distribution prior to and during the pandemic (W=3, Mean Diff =-91.22, Z=2.3102, p<0.05). The gender distribution before and during the pandemic was not significantly different (χ^2^=2.666, df=1, p=0.103). During both periods, more female than male patients visited the facility; however, overall, the percentage of female patients reduced while the males increased during the pandemic. Prior to the pandemic, 90.2% of patients from the Nairobi conurbation visited the facility, as compared to 96.5% during the pandemic. Significantly fewer patients came from outside the conurbation during the pandemic (χ^2^=98.626, df=1, p=0.000). Although the highest proportion of new patients attended the diagnosis clinic followed by the paediatric dentistry clinic both before and during the pandemic, the actual numbers were fewer during the pandemic (oral diagnosis - 1793, 50.8% compared to 1561, 52.8% and paediatric dentistry - 1054, 29.9% compared to 705, 23.8%). Although not significantly different (W=16.5, Mean Diff=-13.5, Z=-0.21, p>0.05), prior to the pandemic, pain and mobility were the most frequent complaints as compared to during the pandemic when pain (38.5% to 46.2%) and swelling (14.1% to 21.2%) were most frequent.

The reduction in the number of patients was similar to trends observed in India [4], China [5], Turkey [6] and the USA [7]. Although there was little variation in the gender distribution, a slight decrease in the proportion of females and an increase among male patients was also witnessed in China [5] and Saudi Arabia [8]. Increased dental-related emergencies were also observed in Nepal [9] with reports of increased reliance on pharmaceutical management of dental conditions in Australia [10].

## Conclusion

We found a decline in the number of new patient visits at the University of Nairobi Dental Hospital during the COVID-19 pandemic, with significantly fewer patients from outside the Nairobi conurbation. The proportion of presenting complaints of pain and swelling were higher during the pandemic. To minimize patient suffering and reduce emergency incidents during such extraordinary circumstances, policies should ensure continued access and utilization of oral health services, patient education and oral health promotion. Clinical training methodologies should promote relevant aspects of asynchronous learning.

## References

[1] Ministry of Health, Kenya (2020). Interim Guidelines on Management of COVID-19 in Kenya - COVID-19. Infection Prevention and Control (IPC) and Case Management.

[2] Otieno BO, Kihara EN, Mua BN (2020). Infection Control Practices Among Private Practicing Dentists in Nairobi During the Pre-coronavirus Disease 2019 Period. Front Oral Health.

[3] Ogunleye OO, Basu D, Mueller D, Sneddon J, Seaton RA, Yinka-Ogunleye AF (2020). Response to the Novel Corona Virus (COVID-19) Pandemic Across Africa: Successes, Challenges, and Implications for the Future. Front Pharmacol.

[4] Madi M, Kumar M, Varchas P, Vineetha R, Pentapati KC (2021). Changing trends in the outpatient dental visits during the COVID-19 pandemic in a tertiary care hospital. Saudi J Biol Sci.

[5] Guo H, Zhou Y, Liu X, Tan J (2020). The impact of the COVID-19 epidemic on the utilization of emergency dental services. J Dent Sci.

[6] Üstün N, Akgöl BB, Bayram M (2021). Influence of COVID-19 pandemic on paediatric dental attendance. Clin Oral Investig.

[7] Choi S, Simon L, Basu S, Barrow J (2021). Changes in dental care use patterns due to COVID-19 among insured patients in the United States. J Am Dent Assoc.

[8] Meisha DE, Alsolami AM, Alharbi GM (2021). Social determinants of seeking emergency and routine dental care in Saudi Arabia during the COVID-19 pandemic. BMC Oral Health.

[9] Dixit P, Dixit S, Dahal S, Poudel P, Roy D, Manandhar N (2020). Pattern of dental problems among patients visiting a dental hospital during covid-19 pandemic. Kathmandu Univ Dent J.

[10] Mian M, Teoh L, Hopcraft M (2021). Trends in Dental Medication Prescribing in Australia during the COVID-19 Pandemic. JDR Clin Trans Res.

